# A Novel Approach to the Symptomatic Management of Chronic Megacolon

**DOI:** 10.1155/2021/8820724

**Published:** 2021-01-25

**Authors:** Michelle J. Ward

**Affiliations:** Cairns Base Hospital, Australia

## Abstract

**Background:**

Chronic megacolon is a rare condition which primarily occurs in patients with autonomic dysfunction of a variety of causes. Its management is often challenging and people with chronic megacolon often suffer from abdominal distension, pain, and malabsorption. Given the struggles clinicians experience in managing these patients long term, this case study provides an example of an alternate strategy for the symptomatic management of chronic megacolon. *Case Description*. An 80-year-old male with early Parkinson's disease developed megacolon following a basal ganglia stroke. He had a protracted hospital stay over 6 months due to malabsorption requiring total parenteral nutrition and electrolyte disturbances. A trial of subcutaneous neostigmine was unsuccessful, so patient underwent a trial of intermittent rectal tube decompression which improved his symptoms and malabsorption. This technique was then taught to the patient's wife until she was confident performing this herself. With continuation of decompression approximately every three days, the patient was able to return to oral nutrition and no longer required ongoing electrolyte replacement. He was able to be discharged into the community with significant improvement in his quality of life.

**Conclusion:**

This is the first report to suggest the benefit of intermittent rectal tube decompression in the community for the long-term management of chronic megacolon. Further prospective studies should evaluate the potential for this strategy to be implemented in a wider cohort of patients who are not responsive to existing treatments for chronic megacolon.

## 1. Introduction

Chronic megacolon is a rare condition, whose causation is variable, and management is difficult. In adults, chronic megacolon is most commonly idiopathic, although in cases where a cause can be identified it may be broadly be grouped into systemic and central neurological entities. Common systemic associations include diabetes, hypothyroidism, paraneoplastic syndrome, autoimmune conditions, and viral infections, while neuropathic conditions include Parkinson's, stroke, encephalopathy, orthostatic hypotension, and Shy-Drager syndrome. Given the rarity and heterogeneity of the condition, there is no standardised approach to management and therefore treatment has largely been driven by low-powered studies and anecdotal treatments. In the long term, the condition almost inevitably results in discomfort, malnutrition, and compromise to quality of life. [1–6]

## 2. Case Presentation

An 80-year-old man with early Parkinson's disease suffered a basal ganglia stroke in November 2017. He was admitted under the geriatric team who commenced him on poststroke treatment and rehabilitation. Over a period of three weeks, he began to develop increasing abdominal distension with associated decreased oral intake, wasting, and electrolyte derangements including refractory hypokalaemia. He was consulted on by the Acute Surgical Unit who organised an abdominal X-ray which showed marked bowel distension and proceeded to manage him with rectal tube decompression and aggressive electrolyte replacement. The rectal tube remained in situ for approximately 1 week before being removed, during which time the patient was able to resume a normal oral intake and participate in regular physiotherapy. However, within 1 week of rectal tube removal, he again became distended with associated decreased oral intake and electrolyte derangement. He once again had a rectal tube inserted with subsequent improvement and was consulted on by the renal team to further investigate whether his hypokalaemia was the primary issue or a consequence of his chronic megacolon. They failed to find anything significant, and upon removal of the rectal tube, the patient once again relapsed.

At this point, the patient was referred to the colorectal team for ongoing consultation.

Flexible sigmoidoscopy was performed to exclude any evidence of mechanical obstruction, and a lengthy discussion was had with all involved regarding the appropriateness for surgical resection or caecostomy. Given the patient's malnutrition and poor functional status, this was not deemed to be in the patient's best interests, and a decision was made to trial the patient on subcutaneous neostigmine which has been shown to be of benefit in a number of small case series on acute megacolon. He was commenced on 0.25 mg subcutaneous neostigmine four times a day with twice daily microlax enemas. This produced some effect over the period of a week with improvements in abdominal distension, appetite, and activity. As such, a decision was made to begin reducing the dose as most previous studies have used neostigmine only for a few days before weaning or ceasing. Upon reducing the frequency to three times a day, the patient began to experience some increased bloating but to a much lesser extent than previously, and he was continued on this dose for a further week.

A decision was made with the patient, his wife, and the treating team to commence discharge planning, but it was decided that it would be unlikely for the patient to be able to continue on subcutaneous neostigmine on discharge. A small summary series looking into the use of pyridostigmine was reviewed, and it was thought this may provide a reasonable alternative for the outpatient setting, and the patient was transferred from neostigmine to pyridostigmine. Unfortunately, over the next 6 days, the patient once again became increasingly distended and unable to eat. He developed increasing abdominal pain and difficulty breathing and a decision was made to terminate the trial with immediate rectal tube decompression and resumption of subcutaneous neostigmine. However, the neostigmine was noted to have less effect than on initial commencement. Surgical options such as caecostomy or colostomy were considered; however, given the patient's frailty, there were significant concerns regarding the patient's ability to tolerate an anaesthetic resulting in other options being sought.

Up until this point, the only intervention that seemed to have a consistent effect seemed to have been rectal the insertion; however, this was only undertaken when the patient was very distended with difficulty breathing and unable to manage any oral intake, and the idea of prolonged rectal tube decompression was dismissed due to the risk of rectal ulceration and the difficulties with long-term management. This however promoted the idea of a different approach. We have had long-term robust data in urology into the concept of intermittent self-catheterisation for urinary retention, and it was thought that the same concept could potentially be extrapolated to intermittent rectal tube decompression for management of megacolon. The patient himself was unable to self-insert a rectal tube, but up until this point, the patient's wife had been administering twice daily microlax enemas. As such, we broached the idea with the patient and his wife whether she would be willing to learn rectal tube insertion for decompression. Having been in hospital for more than 5 months at this point, they were willing to consider any options that may give them an opportunity for discharge into the community with some degree of symptom control and they readily agreed.

Over the next 3 days, we did daily rectal tube education with the patient and his wife with good decompression of both gas and faeces. A decision was made to use a 28 or 30Fr Foleys catheter as the equipment was easily available, and the soft-tipped tube minimised the risk of accidental rectal injury. The duration between rectal tube insertion was then gradually increased with insertion based on patient symptoms of discomfort and decreased appetite to the point where insertion was occurring approximately every three days. The patient reported subjective improvement of symptoms and also optimism at the prospect of potential discharge, while the patient's wife felt confident managing rectal tube insertion, drainage, and removal. There was also an associated decrease in supplemental potassium replacement, increased weight, and increased functional status from fully dependent to partially dependent. After a six month stay in hospital, the patient was finally able to be discharged to a nursing home with the facility providing equipment for ongoing decompression and ongoing follow up in the colorectal outpatient department.

## 3. Discussion

Chronic megacolon is a debilitating condition for both the sufferer as well as having significant impacts on their families and teams associated with their care. The first step is diagnosis of the condition. Imaging, endoscopy, and treating causes of acute megacolon are essential in excluding a mechanical obstruction such as cancer or volvulus and establishing the chronicity of the condition. Secondly, while subcutaneous neostigmine has been shown to be of some benefit in the short term management of acute megacolon, its role in chronic megacolon is much less convincing as there seems to be less of an effect with longer-term use. The role of pyridostigmine in this case was not equivalent to that of subcutaneous neostigmine although the role of reducing impact over time may have also somewhat contributed to the lack of effect in this case. And finally, intermittent rectal tube decompression may play a role in the long-term symptomatic management of patients with this condition.

## 4. Conclusion

Intermittent rectal tube decompression may potentially have a role in long-term symptomatic management in patients with chronic megacolon.
